# Growth factors, silver dressings and negative pressure wound therapy in the management of hard-to-heal postoperative wounds in obstetrics and gynecology: a review

**DOI:** 10.1007/s00404-015-3709-y

**Published:** 2015-04-12

**Authors:** Paweł Jan Stanirowski, Anna Wnuk, Krzysztof Cendrowski, Włodzimierz Sawicki

**Affiliations:** Department of Obstetrics, Gynecology and Oncology, II Faculty of Medicine, Mazovian Bródno Hospital, Medical University of Warsaw, Warsaw, Poland; Mazovian Bródno Hospital, Kondratowicza 8, 03-242 Warsaw, Poland

**Keywords:** Gynecology, Growth factor, Obstetrics, Negative pressure wound therapy, Platelet-rich plasma, Silver dressing, Vacuum-assisted closure

## Abstract

**Purpose:**

The last two decades witnessed the development of numerous innovative regimens for the management of patients with abnormally healing and infected wounds. Growth factors, negative pressure wound therapy (NPWT) and antiseptic dressings containing silver are examples of methods with best documented efficacy, being widely used in the treatment of acute and chronic post-traumatic wounds, burns and ulcers of various etiology. As far as obstetrics and gynecology are concerned, prevention and treatment of infected, hard-to-heal postoperative wounds is of crucial importance. This article reviews the available literature to discuss the possibilities for use, efficacy and cost-effectiveness of growth factors, NPWT and silver dressings in the treatment of difficult-to-heal postsurgical wounds in obstetrics and gynecology.

**Materials and methods:**

An extensive search of the English and Polish literature via PubMed and EMBASE databases was undertaken for articles published between January 1960 and April 30, 2014 to identify articles that described and assessed use, efficacy and cost-effectiveness of growth factors, silver dressings and NPWT in patients with hard-to-heal postoperative wounds following obstetric or gynecological surgery.

**Conclusions:**

Literature review regarding the use of growth factors, NPWT and silver dressings suggests that these methods may play an important role in the management of wounds after invasive obstetric and gynecological procedures. Obese patients, patients after vulvectomy or prior radiation therapy may benefit most, however, due to non-numerous randomized reports, prospective studies on the use of above-mentioned methods in the treatment of postsurgical wounds following obstetric and gynecological interventions are required.

## Introduction

Postoperative wound healing complications constitute an important medical and socioeconomic problem worldwide. Despite the fact that the risk factors responsible for the impaired healing process were identified and the continuously increasing medical knowledge in the fields of tissue engineering, molecular biology and microbiology facilitated the development of numerous new recommendations and methods for management, in many cases the available options for successful treatment of postoperative wounds remain limited. Chronic, difficult-to-heal wounds occurring as complications of various disorders associated with insufficient oxygen and nutrient supply to the cells are potential sources of infection and lead to necrosis of the surrounding tissues. In consequence, non-treated or inappropriately treated postsurgical wounds may separate, lead to formation of fistulas, or become sites of origin for systemic infections. Patients are exposed to risk of further complications and hospitalization time extends resulting in increased treatment costs. Treatment prolongation affects also the quality of life and psychosocial functioning of patients with impaired wound healing. Considering the arguments above, appropriate management of postoperative wounds is currently one of the priorities for the majority of invasive medical disciplines.

Obstetrics and gynecology are fields in which the issues associated with wound healing are particularly relevant. According to the literature data, the rate of infected, hard-to-heal wounds for the two most common interventions in obstetrics and gynecology, i.e., cesarean section and abdominal hysterectomy, is 1.8–12.2 % with 0.3–1.2 % of cases being associated with subsequent wound dehiscence [1–12]. The less common procedure of vulvectomy with or without accompanying inguino-femoral lymphadenectomy is characterized by 12.5–39 % of cases of wound healing disorders [13].

Recently formed concepts for the treatment of chronic and difficult-to-heal wounds assume full comprehensiveness of therapy. This involves the need for systemic treatment being undertaken simultaneously with direct therapeutic activities at the site of the injury. The goal of the systemic treatment is to ensure conditions that promote healing by elimination of risk factors responsible for the abnormal course of the wound healing process, including infections, obesity, malnutrition, anemia and nicotinism, as well as efficient treatment of concomitant diseases such as diabetes, malignancy or autoimmune diseases.

According to the TIME strategy (tissue management, infection and inflammation control, moisture imbalance, epithelial advancement) developed by the European Wound Management Association in 2004, topical wound treatment involve the sequential stages of wound debridement, infection control, maintaining appropriate moisture and stimulation of epithelialization [14]. The goal of wound debridement is to clear the wound bed of foreign bodies, necrotic tissue and excessive exudate that constitute potential sources of infections while also hindering the development of granulation tissue and epithelial edge advancement. Debridement may be either invasive using surgical instruments, or conservative, involving mechanical (hydrosurgery, low-frequency ultrasound), enzymatic (collagenase), autolytic (hydrogels, honey), chemical [antiseptics, i.e., octenidine, chlorhexidine, silver, polyhexamethylene biguanide (PHMB)] or larval methods [15]. Reduction of infection and the indirectly associated inflammation control are achieved by administration of prophylactic doses of antibiotics in the perioperative period, postoperative use of antiseptic dressings (silver, honey, iodine, or PHMB) and lavasepsis consisting in cleansing the wound with antiseptics prior to each dressing change [15, 16]. Maintaining appropriate moisture balance, exudate management and promotion of regeneration processes, such as epithelialization, are tasks where crucial role is played by biologically active dressings, and recently also by negative pressure techniques [15].

The concept of active dressings was initiated and developed in 1962 by Winter, who demonstrated that moist dressing environment accelerated re-epithelialization and wound healing by a factor of two as compared with traditional dry dressings [17]. Studies conducted by Winter’s successors confirmed his idea and led to the development of an “ideal dressing” model. According to the model, topical compress should not only provide for external protection of the wound, but mainly stimulate the regeneration processes, e.g., by ensuring active wound debridement, maintaining appropriate moisture with the appropriate pH, gas exchange and thermal regulation within the wound bed [18]. The “ideal dressing” should also absorb excess exudate while causing no allergic reactions and being easy to place and remove so as not to damage the wound edges upon replacement. As a result, departure from the traditional methods of covering wounds with dry gauze dressings that have no other role than protective can be observed.

The proposed dressing model became a basis for the research of new, efficient methods of wound management. The most innovative methods include growth factors, platelet-rich plasma (PRP) and derivatives, negative pressure wound therapy (NPWT), and antiseptic silver dressings. Numerous reports are available to evidence the efficacy of these methods in the management of postoperative wounds, e.g., in general, plastic and trauma surgery [19–22]. As far as obstetrics and gynecology are concerned, the number of published studies on the usefulness of the growth factors, NPWT and silver dressings in the treatment of hard-to-heal and infected wounds is still very low and insufficient. Considering the number of procedures performed within the pelvis minor region in females as well as the continuously increasing number of patients undergoing cesarean section, analysis of the usefulness, efficacy and cost-effectiveness of these methods for the treatment of postoperative wounds in obstetrics and gynecology appears to be justified.

## Methods

### Search strategy

A review of the English and Polish literature was undertaken for articles published between January 1960 and April 30, 2014 to identify articles that described and assessed use, efficacy and cost-effectiveness of growth factors, silver dressings and negative pressure wound therapy in patients with hard-to-heal (infected, dehisced) postoperative wounds following obstetric or gynecological surgery.

Studies were identified via PubMed and EMBASE databases using keywords: “growth factor,” “platelet rich plasma,” “platelet gel,” “silver dressing,” “negative pressure wound therapy” or “vacuum assisted closure” combined with “wound” and “obstetrics,” “gynecology,” “hysterectomy,” “vulvectomy” or “cesarean section” by two authors (PS, AW) independently. The reference lists of retrieved articles were reviewed to locate additional studies.

### Study selection

**Fig. 1:**
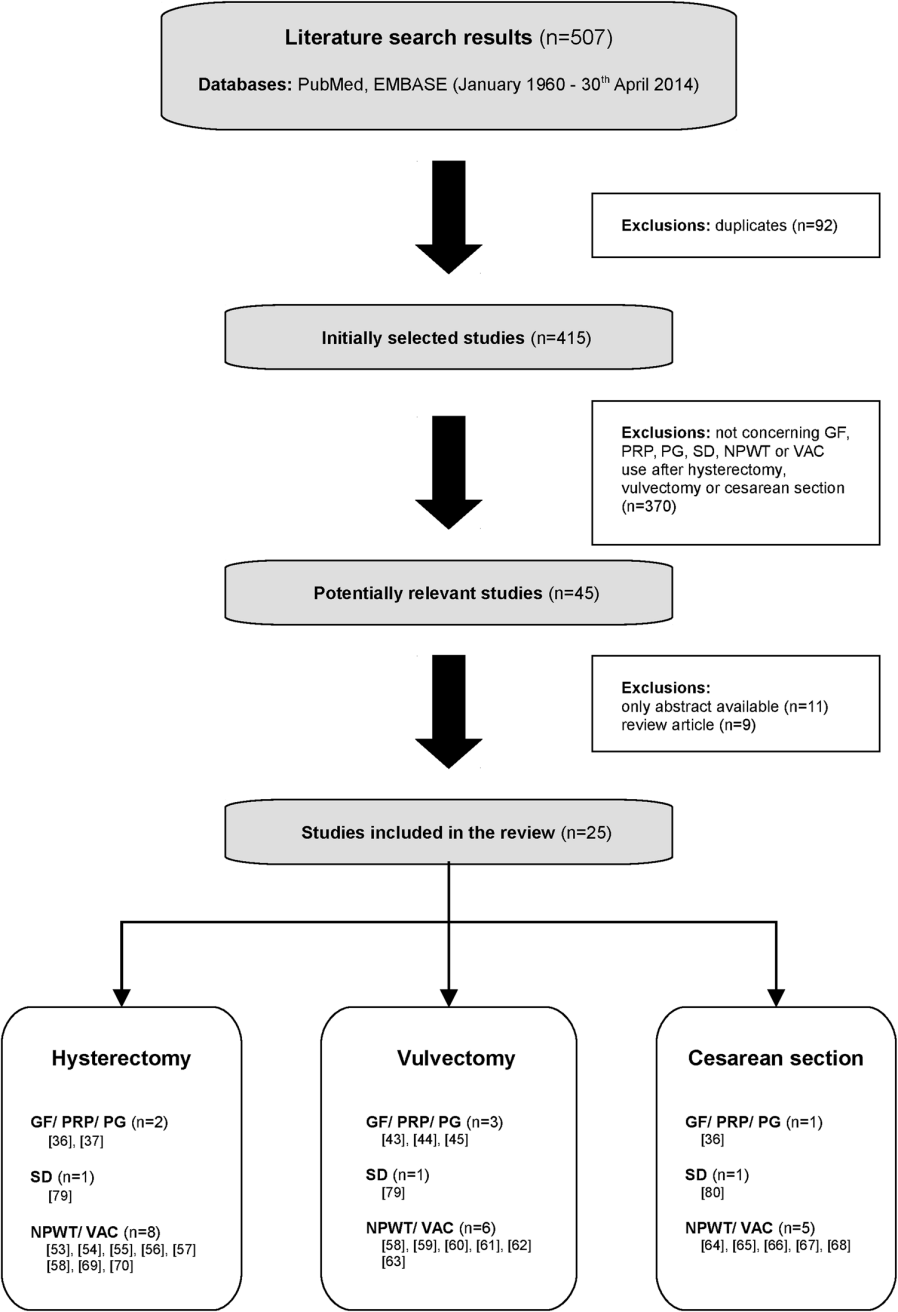
Diagram detailing literature search and study inclusion/exclusion criteria. *GF* growth factor, *PRP* platelet-rich plasma, *PG* platelet gel, *SD* silver dressing, *NPWT* negative pressure wound therapy, *VAC* vacuum-assisted closure

A total of 507 potentially useful publications were identified including 92 duplicates (*n* = 415). Only studies describing growth factor, platelet-rich plasma, platelet gel, silver dressing, negative pressure wound therapy or vacuum-assisted closure use after hysterectomy, vulvectomy or cesarean section were considered relevant (*n* = 45). Publications eligible for the study included full text: randomized controlled trials, cohort studies, case report and case series studies. Abstracts, conference supplements and review articles were excluded. A total of 25 studies were finally retained and reviewed in detail (Fig. 1).

### Study analysis

Following data were collected: study design, patient population, surgical intervention, method of treatment, regimen, outcomes, follow-up, complications and statistical analysis. The characteristics of selected publications are summarized in Table 1.

**Table 1:** Characteristics of studies included in the review

No.	References	Study design	Patient population/surgical intervention	Method of treatment	Regimen
1.	Shackelford et al. [36]	RCT	24 patients with wound separation after CS or benign abdominal gynecologic procedures; *n* = 12 treatment group, *n* = 12 control group	0.01 % rhPDGF-BB gel or placebo	Topical daily application
2.	Fanning et al. [37]	Prospective non-randomized	110 patients after major gynecologic, surgery; *n* = 55 study group, *n* = 55 historical control group	Surgery + APTG or surgery alone	Direct postoperative application to the surgical site
3.	Morelli et al. [43]	Retrospective	25 patients after RVIFL; *n* = 10 study group, *n* = 15 control group	Surgery + PG or surgery alone	Direct postoperative application to the surgical site
4.	van Lindert et al. [44]	Prospective non-randomized	22 patients after RVIFL; *n* = 11 study group, *n* = 11 historical control group	Surgery + rhG-CSF or surgery alone	300 μg/day subcutaneously 1 day before surgery, on the day of surgery and daily for 5 consecutive days after surgery
5.	Uyl-de Groot et al. [45]	RCT	40 patients after RVIFL; *n* = 20 study group, *n* = 20 control group	Surgery + rhG-CSF or surgery + placebo	300 μg/day subcutaneously 1 day before surgery, on the day of surgery and daily for 7 consecutive days after surgery
6.	Argenta et al. [53]	Case series	3 patients: P1: subcutaneous dehiscence after TAH and herniorrhaphy P2: wound dehiscence after TAH + BSO for endometrial cancer P3: wound defect and enterocutaneous fistula after exploratory laparotomy for ovarian cancer and relaparotomy for small bowel perforation	VAC	P1, P2, P3: intermittent negative pressure of 125 mmHg; dressing replacement every 48 h
7.	Miller et al. [54]	Case report	59 year old, moderately obese patient with wound dehiscence after abdominal hysterectomy	NPWT	Negative pressure of 80 mmHg for 6–8 h daily; 3 dressing replacements per week
8.	Stannard et al. [55]	Case series	2 patients P1: BMI 50 kg/m^2^ after TAH + BSO for endometrial cancer P2: BMI 60 kg/m^2^ after TAH + BSO for endometrial cancer	Prophylactic NPWT	Continuous negative pressure of 125 mmHg for 4 days after surgery
9.	Gourgiotis et al. [56]	Case report	67 years old patient, BMI 41 kg/m^2^, fascial dehiscence and skin defect after TAH + BSO for endometrial cancer and relaparotomy for sigmoid colon perforation, abdominal compartment syndrome	VAC	Dressing replacement every 48 h
10.	Lavoie et al. [57]	Case report	73 year old patient, BMI 50 kg/m^2^, with wound hematoma and adipose tissue necrosis after TAH + BSO for endometrial cancer	NPWT with gauze filler	NA
11.	Schimp et al. [58]	Retrospective	27 patients with complex wound failures after TAH + BSO (*n* = 14), RV with or without IFL (*n* = 5), skin or myocutaneous grafting (*n* = 3), parastomal herniorrhaphy (*n* = 2), retroperitoneal lymph node dissection (*n* = 2), drainage of gluteal abscess (*n* = 1)	VAC	Negative pressure of 50–125 mmHg applied directly after reoperation (*n* = 4) or after wound failure (*n* = 23); dressing replacement every 48 h
12.	Narducci et al. [59]	Retrospective	54 patients after RV or wide local vulvectomy with or without IFL and/or myocutaneous grafting; study group (*n* = 30), control group (*n* = 24)	VAC or conventional care (perineal irrigation and air drying)	Continuous negative pressure of 100–125 mmHg applied within 24 h of surgery; dressing replacement every 48–72 h
13.	Riebe et al. [60]	Case series	2 patients P1: after palliative tumor debulking with IFL for locally advanced vulvar cancer P2: after RVIFL for locally advanced vulvar cancer	Polypropylene mesh implantation + prophylactic VAC	Continuous negative pressure of 125 mmHg applied directly after surgery; dressing replacement every 48–72 h
14.	Shvartsman et al. [61]	Case report	41 year old patient after vulvectomy for recurrent Paget’s disease	VAC + split-thickness skin graft	Negative pressure of 50–125 mmHg applied directly after surgery and skin grafting; dressing replacement every 48 h
15.	Dainty et al. [62]	Case series	7 patients including 4 patients after vulvectomy for Paget’s disease (*n* = 2) or hidradenitis suppurativa (*n* = 2)	Fibrin tissue adhesives + VAC + split-thickness skin graft	Intermittent negative pressure of 100 mmHg applied directly after surgery and skin grafting for 3–4 days
16.	Piovano et al. [63]	Case report	58 year old patient after RVIFL for syringoid eccrine carcinoma	VAC	NA
17.	Bullough et al. [64]	Prospective non-randomized	50 patients after CS with BMI >35 kg/m^2^	Prophylactic NPWT	Direct postoperative application to the surgical site for 7 days
18.	Mark et al [65]	Retrospective	63 patients after CS with BMI >45 kg/m^2^; *n* = 21 study group, *n* = 42 control group	Prophylactic NPWT or standard surgical dressing	Direct postoperative application to the surgical site
19.	Nissman et al. [66]	Case report	27 year old patient after CS; BMI = 32 kg/m^2^; necrotizing fasciitis	Surgery + NPWT	NA
20.	Durai et al. [67]	Case report	31 year old patient after CS; necrotizing fasciitis	Surgery + VAC	Negative pressure therapy for a minimum of 2 weeks
21.	Ottosen et al. [68]	Prospective	10 patients including 4 patients with wound infection/rupture after CS	NPWT	Negative pressure therapy for a minimum of 2 days in an outpatient setting
22.	Lewis et al. [69]	Retrospective	Historical cohort of 431 patients after laparotomy for endometrial cancer; 134 patients with wound complications (31 %)	Prophylactic NPWT or routine care	Direct postoperative application to the surgical site; negative pressure therapy for 4–7 days
23.	Beral et al. [70]	Case report	67 year old patient with superficial wound dehiscence after TAH + BSO for ovarian cancer	VAC	Negative pressure therapy for 15 days; dressing replacement every 72 h; portable VAC device for several weeks
24.	Sioma-Markowska U. [79]	Case series	3 patients P1, P2: wound infection after RV P3: wound infection after abdominal hysterectomy	Autolytic debridement + lavasepsis + metallic-coated SD	P1, P2, P3: dressing replacement every 48–72 h
25.	Connery et al. [80]	Retrospective	72 patients after CS; *n* = 36 study group, *n* = 36 control group	Metallic-coated SD or gauze pad	Direct postoperative application to the surgical site

No.	References	Outcomes	Follow-up	Complications	Statistical analysis
1.	Shackelford et al. [36]	29 ± 14 days of therapy in treatment group vs. 47 ± 24 days in control group (*p* = 0.057); complete wound closure after 35 ± 15 days of therapy in treatment group vs. 54 ± 26 days in control group (*p* = 0.05); more rapid wound closure in treatment group	3 patients lost to follow-up (1 in treatment group, 2 in control group)	NA	Wilcoxon rank sum test, Fisher exact test, wound closure rates evaluated with Kaplan–Meier survival estimate
2.	Fanning et al. [37]	Significant reduction in pain on postoperative day 0 and 1 (*p* ≤ 0.001); significant reduction in total dose of morphine (*p* = 0.02)	Follow-up on day 7 and 28 postoperatively; no one lost to follow-up	No adverse effects observed	Chi-square test, Fisher exact test, Student *t* test
3.	Morelli et al. [43]	Significant decrease in wound infection, necrosis and breakdown rates (*p* = 0.032; *p* = 0.096; *p* = 0.048, respectively); significant decrease in postoperative fever rate and hospital stay (*p* < 0.001); complete wound closure after 24 days of therapy in study group vs. 93 days in control group (*p* < 0.001)	NA	NA	Chi-square test, Fisher exact test, Student *t* test
4.	van Lindert et al. [44]	Primary wound healing observed in 69.7 % of vulva and groin wounds in study group vs. 25 % in control group; major wound breakdown observed in 6.1 % of vulva and groin wounds in study group vs. 50 % in control group	Follow-up on day 5 and 10 postoperatively; no one lost to follow-up	Major wound breakdown or skin separation observed in 6.1 % and 15.1 % of vulva and groin wounds, respectively	NA
5.	Uyl-de Groot et al. [45]	No significant differences observed with respect to wound infection and primary wound healing rates; no significant differences observed with respect to quality of life; total treatment cost in study group EUR 15,951 vs. control group EUR 12,430	Follow-up on day 30 and 56 postoperatively; 1 patient in study group excluded; 3 patients in study group lost to follow-up	Nausea (1 patient); elevated liver enzymes (3 patients)	Student *t* test, Mann–Whitney test
6.	Argenta et al. [53]	P1: significant decrease in wound volume after 14 days of therapy; complete wound closure 4 weeks postdischarge P2: significant decrease in wound volume after 10 days of therapy; complete wound closure P3: significant decrease in wound volume after 13 days of therapy, fistula closure on day 7 of therapy; complete wound closure In all patients enhanced granulation tissue formation was observed within the first 48 h of VAC therapy	4–13 months	P1: none P2: none P3: none	NA
7.	Miller et al. [54]	Wound closure after 3 months; no analgesics required	3 months	NA	NA
8.	Stannard et al. [55]	P1: complete wound closure after 4 weeks P2: uncomplicated wound healing	P1: 4 weeks postoperatively P2: 4 days postoperatively	P1: superficial skin separation on postoperative day 14	NA
9.	Gourgiotis et al. [56]	Complete fascial closure after 21 days	3 months	None	NA
10.	Lavoie et al. [57]	7 days of therapy; patient discharge after 36 days following debridement; complete wound closure after 82 days following debridement; no skin grafting required	2 months	None	NA
11.	Schimp et al. [58]	96 % reduction in the median size of wound defect; median VAC therapy length 32 days (range 3–88 days); at the time of last contact 26 of 27 patients presented complete wound healing	Median follow-up 52 days (range 0–270 days)	Bleeding (1 patient); 67 % of patients complained of pain during dressing change	NA
12.	Narducci et al. [59]	Median VAC therapy length 11 days (range 6–38 days); complete wound closure after 44 ± 18 days of therapy in study group vs. 60 ± 29 days in control group (*p* = 0.0175); no significant difference in length of hospital stay	Median follow-up 19.1 ± 11.2 months	Partial necrosis of myocutaneous flap (1 patient); frequent vestibular stenosis	NA
13.	Riebe et al. [60]	P1: granulation tissue reached skin level on 32 postsurgical day P2: granulation tissue reached skin level on 39 postsurgical day	10 months	None	NA
14.	Shvartsman et al. [61]	16 days of therapy; successful graft adherence	12 months	None	NA
15.	Dainty et al. [62]	~90 % graft-take rate (3 patients) ~60 % graft-take rate (1 patient)	follow-up on day 7 postoperatively	Keloid formation and wound contracture (1 patient)	NA
16.	Piovano et al. [63]	4 weeks of therapy; complete wound healing	12 months	None	NA
17.	Bullough et al. [64]	No SSI observed; no hospital readmissions	~1 month	Allergic reaction (2 patients)	NA
18.	Mark et al [65]	Decrease in wound complication rate 0 % study group vs. 10.4 % control group (*p* = 0.15)	1 month	None	Chi-square test, Student *t* test
19.	Nissman et al. [66]	Complete wound healing	NA	None	NA
20.	Durai et al. [67]	Complete wound healing	6 weeks	None	NA
21.	Ottosen et al. [68]	Treatment experienced as effective; high level of dependency on the equipment at the beginning of therapy; experience of embarrassment; importance of relatives help and support	Median follow-up 4 weeks (range 2–8 weeks) postdischarge	NA	Ricoeur method
22.	Lewis et al. [69]	Mean overall cost of incision care following NPWT $509 vs. routine care $613	NA	Risk of skin blistering following NPWT estimated at 20 %	Assumption of 50 % reduction in risk of wound complication following NPWT
23.	Beral et al. [70]	Wound healing with purulent discharge	NA	Retained pieces of foam in the wound area	NA
24.	Sioma-Markowska U. [79]	P1, P2: complete wound healing P3: complete wound healing after 14 days Treatment outcomes experienced as positive by all patients	NA	P1: none P2: none P3: none	NA
25.	Connery et al. [80]	No significant difference observed with respect to SSI rate between both groups	1 month; no one lost to follow-up	SSI (2 patients in study group vs. 2 patients in control group)	Chi-square test, Student *t* test

*RCT* randomized controlled trial, *CS* cesarean section, *rhPDGF-BB* recombinant human platelet-derived growth factor BB, *NA* not applicable, *APTG* autologous platelet tissue graft, *RVIFL* radical vulvectomy with inguino-femoral lymphadenectomy, *PG* platelet gel, *rhG-CSF* recombinant human granulocyte colony-stimulating factor, *TAH* total abdominal hysterectomy, *TAH* *+* *BSO* total abdominal hysterectomy with bilateral salpingo-oophorectomy, *VAC* vacuum-assisted closure, *NPWT* negative pressure wound therapy, *BMI* body mass index, *SSI* surgical site infection, *SD* silver dressing

## Growth factors in the management of hard-to-heal postoperative obstetrical and gynecological wounds

The modern concept of an “ideal dressing” assumes that the dressing should not only play a protective role or provide appropriately moist conditions, but also directly stimulate cellular regeneration. Starting from the mid-1980s, many researchers focused on cellular growth factors and opportunities of their use in the treatment of chronic wounds [23]. The growth factors being simultaneously cytokines and biologically active peptides of auto- and paracrine activity are characterized by pleiotropic effect on the course of the healing process. By binding membrane receptors of the target tissues, the growth factors trigger intracellular signaling pathways and stimulate cellular proliferation, differentiation and migration [19, 24, 25]. Importantly, the fact that growth factors do not penetrate the cell interior and thus do not directly interact with the nucleus eliminates the potential risk of mutagenic effects and neoplasia.

Due to their wide spectrum of activities and the multitude of functions, attempts are made to use growth factors in the treatment of various disorders and diseases. This mostly pertains to such disciplines as general surgery, maxillofacial surgery, plastic surgery, orthopedics and sports medicine as a consequence of the invasive nature and related necessity to repair damaged tissue structures [19, 20, 23, 26].

In 1997, the US Food and Drug Administration (FDA) approved the recombinant human platelet-derived growth factor BB (rhPDGF-BB, becaplermin) in adjunctive treatment of diabetic neuropathic foot ulcers [20, 23]. To date, this is the only cellular growth factor product to be approved for use in chronic wounds and ulcers management on the basis of multicenter, randomized controlled trials. Animal studies revealed that rhPDGF-BB significantly accelerated the healing of wounds, including ischemic, and that one of the possible mechanisms of its action involves reduction of the levels of proinflammatory cytokines: tumor necrosis factor-alpha (TNF-α), interleukin 1-beta (IL-1β) and metalloproteinase 2 (MMP2) and 9 (MMP9) within the wound [27]. Of the remaining growth factors, keratinocyte growth factor (KGF), granulocyte macrophage colony-stimulating factor (GM-CSF) and epidermal growth factor (EGF) were clinically confirmed to be efficient in the treatment of chronic venous ulcers [23]. The beneficial effect of growth factors such as vascular endothelial growth factor (VEGF) and transforming growth factor-beta (TGF-β) on the wound healing process was demonstrated only in animal studies [23]. This small number of controlled, randomized trials conducted in appropriately large group of patients does not permit any definite conclusions regarding the efficacy of growth factors in the treatment of abnormally healing wounds, including postoperative. On the other hand, due to the high costs associated with preparation of recombinant growth factors and with application techniques preventing growth factors from being degraded too early within the administration site, the method will probably not be considered as the first-line treatment of wounds after invasive procedures.

The platelet-rich plasma (PRP) which constitutes an autologous concentrate of thrombocytes in a small volume of plasma comprises an efficient alternative to growth factors. Depending on the technique used PRP is characterized by a two- to sixfold increase in the platelet count [26, 28]. As a result, concentrations of growth factors produced by thrombocytes are increased several times. Most important growth factors found in the PRP include: 3 isomers of PDGF (PDGFαα, PDGFββ, PDGFαβ), TGFβ1, TGFβ2, VEGF and EGF [26, 28]. Due to the low plasma volume, PRP contains also adhesion proteins such as fibrin, fibronectin and vitronectin involved in the extracellular matrix formation and thus being of importance for the wound healing process. Results of in vitro and animal studies revealed the effect of platelet-rich plasma on the migration, proliferation and differentiation of cells involved in the healing process as well as angiogenesis-stimulating properties [28]. The efficacy of PRP was shown to depend mostly on appropriate preparation technique that ensures a possibly highest level of platelets per unit volume without their simultaneous degradation. To date several systems for PRP preparation were developed with only few of them allowing to achieve the required “therapeutic” platelet concentration of ≥1 × 10^6^/µl [26]. The process for preparation of platelet-rich plasma generates much less costs compared to genetic engineering methods used in preparation of recombinant growth factors while the autologous nature of the product eliminates the risk of transmission of viral infections, such as hepatitis virus or HIV infection.

Discussed below are the attempts made hitherto with regard to the use of cellular growth factors and PRP derivatives in the treatment of difficult-to-heal postsurgical obstetrical and gynecological wounds.

### Laparotomy

Laparotomy, or surgical opening of the abdominal cavity, is one of the most commonly performed surgical procedures. Among patients admitted to gynecological wards, main reasons for exploration of the abdominal cavity include benign and malignant tumors within the uterus and/or adnexa, abnormal vaginal bleedings and endometriosis [6, 29]. A preferred method for the management of most of the aforementioned disorders is abdominal hysterectomy being at the same time the most common invasive procedure in gynecological surgery. According to literature data, nearly one in five women is subjected to hysterectomy before the age of 60 [30].

In obstetrics, the abdominal cavity is opened during cesarean section procedure. Depending on the geographical region, the ratio of cesarean sections to the total number of deliveries varies between 15 and 30 %, with a significant upward trend being observed in the developed countries [31, 32].

Both the gynecological surgeries involving laparotomy and the cesarean section are procedures relatively often complicated by impaired postoperative wound healing. In case of abdominal hysterectomy, the percentage of postoperative wound infections is 3.0–12.2 %, with wound dehiscence occurring in 0.3–0.6 % patients [1–7, 29]. Cesarean section is associated with risk of the above-mentioned complications of 1.8–11.3 % and 0.4–1.2 %, respectively [1, 8–12, 33].

Risk factors responsible for abnormal healing of obstetric and gynecological postoperative wounds are similar to those observed in other surgical disciplines. They include i.a. elderly age, obesity, diabetes, malnutrition, infections (chorioamnionitis in case of cesarean section), immunodeficiency, anemia, renal and hepatic insufficiency, nicotinism, prior radiation therapy and intraoperative technical difficulties extending the overall time of procedure [1, 5, 18, 34, 35]. The size and location of the wound, type of materials used for wound closure and presence of drains are also of high importance.

Although being sparse, studies conducted with regard to the use of growth factors after obstetric and gynecological procedures demonstrate their beneficial effect on wound healing [36, 37]. A double-blinded randomized, placebo-controlled trial performed by Shackelford et al. evaluating rhPDGF-BB efficacy in the treatment of separated surgical wounds after cesarean section or benign abdominal gynecologic procedures revealed a significant reduction in time required for complete wound healing in women receiving the recombinant growth factor [36]. Among 11 patients in the study group, daily topical application of 0.01 % rhPDGF-BB gel resulted in the mean time until wound closure of 35 ± 15 days compared to 54 ± 26 days in the placebo group (*p* = 0.05). Taking into account the difference between the time of procedure and the time of wound dehiscence occurrence, the overall treatment time was 29 ± 14 days in the study group and 47 ± 24 days in the control group (*p* = 0.057).

Fanning et al. conducted a prospective non-randomized study evaluating the toxicity of autologous platelet tissue graft—a derivative of platelet-rich plasma, and its efficacy in decreasing postoperative pain in patients after major gynecological surgeries, e.g., laparoscopic-assisted vaginal hysterectomy, laparoscopic-assisted vaginal hysterectomy with laparoscopic lymphadenectomy, abdominal hysterectomy as well as advanced urogynecological procedures requiring multiple repairs [37]. At completion of the surgical procedure and achievement of adequate hemostasis, the researchers applied a pre-prepared and activated autologous platelet tissue graft directly to the surgical site, including the vaginal cuff, parametrium and fascia. No adverse effects of the treatment were observed in the group of 55 patients who received the autologous platelet tissue graft. Pain experienced on the day of surgery and during the first postoperative day assessed using a ten-point visual analog scoring system was significantly reduced in the study group compared to the control group: 2.7 and 2.1 vs. 6.7 and 5.5 (*p* < 0.001), respectively. An indirect consequence of these outcomes was the reduction in total dose of morphine used to relieve postoperative pain during hospitalization from 26 mg in the control group to 17 mg in the study group (*p* = 0.02).

### Vulvectomy

Vulvar cancer is a relatively rare malignancy of female genital organs, accounting for ca. 5 % of all cases [38]. According to data collected in the Polish Register of Cancer, 463 new cases (standardized morbidity ratio of 1.06/100,000) and 270 deaths (standardized mortality ratio of 0.54/100,000) due to vulvar cancer were recorded in 2011 [39]. As a result, vulvar cancer is the 23rd most common malignancy in Polish women while being the 21st most common cause of deaths.

Following publication of results obtained by Way in 1960, radical vulvectomy with bilateral inguino-femoral lymphadenectomy is considered standard treatment for most patients with advanced cancer of the vulva [38, 40]. Due to surgical site location, extent and mutilatory character, procedure is associated with numerous postoperative complications [13]. Abnormal healing of groin wounds leading to wound breakdown is the most common complication of radical vulvectomy at the early stage. According to first clinical observations, wound complications (infection, dehiscence) occurred in 53–85 % of patients undergoing radical surgery of the vulva [41, 42]. Later implementation of three separate incision technique allowed for a marked reduction in this percentage and currently, infections or dehiscence of postoperative wounds are observed in 21.3–39 % and 12.5–39 % of patients subjected to vulvectomy, respectively [13]. Inguino-femoral lymphadenectomy is considered to be the main reason behind the large percentage of wound healing disturbances. Due to the moist and warm groin environment, the dissection of inguinal lymph nodes increases the risk of wound infection while also leading to chronic lymphedema development. The remaining factors that impede the healing process in patients undergoing radical vulvectomy include central or bilateral tumor location, en bloc surgery, extent of lymphadenectomy, presence of lymphocele, resection of the saphenous vein and prior radiation therapy [13].

There are few reports on the use of cellular growth factor products in the treatment of wounds resulting from radical vulvectomy. Retrospective study conducted by Morelli et al. in a group of 25 patients with vulvar cancer at clinical stage IB and II subjected to radical vulvectomy with inguino-femoral lymphadenectomy revealed that application of a platelet gel before the reconstructive phase of surgery accelerates wound healing [43]. In 10 patients in whom platelet gel was used before the reconstructive phase of surgery, a significant decrease in wound infections, necrosis and dehiscence rates was observed as compared to the control group (30 vs. 73.3 %, *p* = 0.032; 20 vs. 53.3 %, *p* = 0.096; 20 vs. 60 %, *p* = 0.048, respectively). Significantly shortened hospital stays and shorter times until complete wound healing were also noted (6.4 vs. 17.6 days, *p* < 0.001 and 23.6 vs. 93.3 days, *p* < 0.001, respectively). In the opinion of the authors, the platelet gel as a reproducible, low-cost and minimally invasive technique comprises an efficient alternative to myocutaneous flaps.

Results similar to those reported by Italian researchers were observed by van Lindert et al. who used the recombinant human granulocyte colony-stimulating factor (r-metHuG-CSF, filgrastim) in women subjected to radical vulvectomy with bilateral inguino-femoral lymphadenectomy [44]. In a pilot study in a group of 11 patients who received filgrastim in the perioperative period (7 days in total) at the daily dose of 300 µg subcutaneously, the authors observed a reduction in the rates of postoperative dehiscence of inguinal and vulvar wounds as compared to a historical control group. Out of the total number of 33 wounds in the study group, 69.7 % showed primary wound healing and significant dehiscence was observed in 6.1 % of cases. In the group of patients treated with the standard regimen, the respective values were 25 and 50 %.

The pilot study became a starting point for a multicenter, randomized trial conducted in 2004 to assess the efficacy of filgrastim for wound infections prevention and the effect of the treatment on the quality of life of the patients and the overall treatment costs after radical vulvectomy with inguino-femoral lymph nodes dissection [45]. Similar as in the previous study, filgrastim was administered subcutaneously in the perioperative period at the dose of 300 µg/day for a total of 9 days. The analysis did not confirm a positive effect of G-CSF on the reduction of infected wounds rates. In the group of 16 patients receiving filgrastim, infections of wounds were observed in 57.1 % of cases while primary wound healing was observed in 14.3 %. In the control group of 20 subjects receiving placebo, the respective values were 55.6 and 33.3 %. In addition, no differences were demonstrated with regard to the quality of life of patients in both groups, while the overall treatment costs were higher in the G-CSF group (EUR 15,951 vs. 12,430).

In conclusion, results of studies on the use of growth factors in the treatment of wounds after laparotomy and vulvectomy as discussed above provide no unambiguous answer with regard to the efficacy and usefulness of these agents. Data collected to date suggest a possible beneficial effect of rhPDGF and platelet-rich plasma derivatives in the prevention and treatment of wound complications in patients after procedures characterized by high risk of abnormal wound healing, e.g., due to vulvar cancer; however, costs of such treatment should be analyzed. Randomized controlled trials conducted in appropriately large patient groups are lacking with regard to the use of cellular growth factors in the treatment of difficult-to-heal wounds following obstetric and gynecological surgeries and standardization of procedures for preparation and application of growth factors is required.

## Negative pressure wound therapy—an alternative to the standard regimens of postsurgical obstetrical and gynecological wound management

First reports on the possible use of negative pressure as a method to treat chronic and difficult-to-heal wounds date from the late 1980s. Study conducted by Kostiuchenko et al. demonstrated beneficial effect of vacuum used as supportive therapy to surgical debridement in the management of infected wounds [46]. In a group of 116 patients subjected to experimental treatment, placing a suction pump at the wound surface generating a negative pressure of 100 mmHg for 5–10 min both before and after the debridement procedure, better healing results were observed as compared to the group of 105 patients treated in the standard manner.

The concept of the Russian researchers was confirmed by an animal model study published in 1997 by Morykwas et al. [47]. Application of subatmospheric pressure of 125 mmHg contributed to a fourfold increase in blood flow within the wound bed as well as to significant reduction in the tissue bacterial counts within the wound area from 10^8^/g of tissue to 10^5^/g of tissue after 4 days of treatment. Similar result in the control group was noted after 11 days of treatment (*p* < 0.05). In addition, a statistically significant acceleration of granulation tissue formation was observed in comparison with the control group—an increase of 63.3 % upon continuous negative pressure and of 103 % upon intermittent negative pressure application (*p* < 0.05).

A study conducted in the same year with the use of the same methodology to assess the clinical efficacy of NPWT in a group of 300 wounds (175 chronic wounds, 94 subacute wounds, and 31 acute wounds) revealed favorable response to the treatment in 296 cases. A reduction in the edema was observed in the wound region while also confirming the previously observed increase in blood flow and enhanced formation of granulation tissue [48].

Today, NPWT is a worldwide-established method for the treatment of chronic and difficult-to-heal wounds with efficacy confirmed by numerous clinical studies [21, 22, 49, 50]. Similar to growth factors, negative pressure affects processes determining proper wound healing by providing moist environment, increasing blood perfusion, accelerating the formation of granulation tissue and removing excess exudate from the site of the injury, thus indirectly reducing the risk of infection [47, 48, 51]. As a result, the list of NPWT indications is being continuously extended. In 1995, FDA approved NPWT as a method to treat acute and chronic post-traumatic wounds and burns were added to the list of indications 7 years later [49].

Over the years, numerous negative pressure systems have been developed. A standard kit includes a porous polyurethane foam, an adhesive sealing film, a drain, a vacuum pump and a container for secretions. Depending on the intended use, the devices may be either stationary or mobile, and the negative pressure may be dosed in either constant or intermittent manner, usually within the range of 50 mmHg down to 150 mmHg [49, 50, 52]. The most recent models of vacuum-assisted closure devices facilitate: ambulatory use, removal of larger quantities of the secretion and irrigation of the wound site with antibiotic solution and/or local anesthetics; antiseptic silver-coated sponges are also used [49, 50, 52].

Contraindications to NPWT include malignant lesions and extensive necrosis in the wound region, osteomyelitis, fistulas and exposed blood vessels, nerves, bones, or organs [50, 52]. Therefore, it is important to remove necrotized tissue from the wound bed and cover the exposed structures with a non-adherent material, e.g., a silicone dressing prior to applying negative pressure. This barrier material would additionally protect the tissues from growing into the polyurethane foam [50, 52]. In cases of wound infections, it is important to provide local and/or systemic treatment with antiseptic dressings, antifungals or antibiotics and similar to other methods used in wound management, treatment of concomitant diseases combined with elimination of factors disturbing normal healing, e.g., by controlling metabolic disorders due to diabetes or malnutrition is an inseparable part of vacuum therapy [50].

Adverse events are rarely observed with NPWT. Those most common include tissue necrosis, fistula formation as well as pain and bleeding accompanying dressing change due to granulation tissue ingrowth into the foam [50, 52]. The latter two may be prevented by the use of interface dressings separating the tissues from the material filling in the wound bed. Other procedures used in pain management involve reduction in suction power by ca. 25 mmHg, saturation of the dressing with 0.9 % sodium chloride or 1 % lidocaine solution 15–30 min before the planned dressing change, covering the wound bed with hydrogels as well as more frequent dressing changes and premedication with analgesic agents [50, 52].

Similar as in the case of growth factors, the number of studies on the use of NPWT in the treatment of difficult-to-heal obstetric and gynecological postsurgical wounds is low. One of the first reports includes a case series description of complex wound failures after major gynecologic procedures by Argenta et al. [53]. Application of vacuum-assisted closure (VAC) device in three patients who had experienced abnormal wound healing during the postoperative period demonstrated good tolerance and high efficacy with regard to granulation tissue formation within the first 48 h since the initiation. No adverse effects of therapy were observed, and satisfactory results of treatment were obtained despite numerous burdens of patients including morbid obesity, diabetes or ongoing chemotherapy. It is noteworthy that in one case the use of subatmospheric pressure resulted in closure of an enterocutaneous fistula considered to be a contraindication to VAC therapy.

Miller et al. reported a clinical case of wound dehiscence in a moderately obese patient subjected to abdominal hysterectomy in whom negative pressure of 80 mmHg applied for 6–8 h daily contributed to complete healing of the wound after 3 months of treatment [54]. During the entire treatment period involving three dressing changes per week, the patient required no analgesics which, according to authors, supports the idea of using lower vacuum levels than generally accepted. In a case series study by Stannard et al., the authors suggested a possibility of a prophylactic use of NPWT directly after the surgery (continuous negative pressure of 125 mmHg for 4 days) to prevent wound infection and breakdown in morbidly obese patients subjected to abdominal hysterectomy [55]. In another case report by Gourgiotis et al. the application of topical VAC therapy in patient with abdominal compartment syndrome and skin defect following major gynecologic surgery reduced the need for fluids and vasopressor agents, prevented fascial retraction and visceral adherence, and finally enabled delayed fascial closure [56]. Lavoie et al. presented effective use of NPWT with gauze filling in the case of extensive adipose tissue necrosis following abdominal hysterectomy [57].

In 2004, Schimp et al. published a report on the efficacy of VAC device in patients subjected to major gynecological procedures, such as total abdominal hysterectomy with bilateral salpingo-oophorectomy and vulvectomy with or without an inguinal lymph node dissection in whom complex wound failures occurred during the postoperative period [58]. A retrospective study included a group of 27 women; 25 diagnosed with malignant tumors of the uterine cervix, endometrium, ovary and vulva. In 23 cases, VAC device was used upon wound dehiscence occurrence (range 0–88 days postoperatively); in the remaining 4 patients vacuum device was placed directly after the reoperation; in 3 women, the dehiscence was located in a previously irradiated area, and wound infection was clinically confirmed in 10 patients. The range of negative pressure used was between 50 and 125 mmHg; dressings were changed in 2-day intervals, occasionally after premedication with oral analgesics. The mean period of vacuum use in the study was 32 (3–88) days; during this time, the authors observed a significant reduction in the size of the wound—96 % reduction as compared to the baseline area. In one case, the treatment was discontinued due to bleeding, while 67 % of the remaining patients complained of pain that accompanied dressing changes. No other complications or treatment-emergent adverse effects were observed. At the time of last visit (mean follow-up: 52 days), 96 % of patients presented complete wound healing.

In a retrospective non-randomized study conducted by Narducci et al. in a group of 54 women subjected to radical vulvectomy or wide local vulvectomy with defect volume larger than 40 cm^3^, inguinal lymphadenectomy and/or myocutaneous flap reconstruction, the authors observed a statistically shorter time until complete wound healing after VAC therapy as compared with the standard management consisting in irrigation of the surgical site with 0.9 % sodium chloride and air drying [59]. Among 30 patients (2 with previous radiotherapy history) in whom constant subatmospheric pressure of 100–125 mmHg was started within the first 24 h after the surgery, the overall time until complete wound healing was 44.4 ± 18.4 days compared to 60.2 ± 28.7 days in a control group of 24 subjects (*p* = 0.0175). The mean duration of therapy involving dressing changes at intervals of 48–72 h performed under local or neuroleptic anesthesia was 11 (range 6–38) days. No statistically significant difference was observed with respect to the mean hospital stay between both groups. Complications of VAC observed by the authors included several cases of vestibular stenosis and one case of partial necrosis of the myocutaneous flap used for vulvar reconstruction.

Riebe et al. presented case series study regarding two patients with locally advanced vulvar cancer who received extensive surgical treatment including tumor debulking and inguino-femoral lymphadenectomy [60]. After polypropylene mesh was implanted over the exposed blood vessels followed by VAC system application, authors observed faster wound healing with lack of complications. Taking into account the fact that exposed vessels, similarly as fistulas, used to be considered as contraindications to VAC therapy initiation these observations provide new evidence regarding possibility to use subatmospheric pressure in the treatment of hard-to-heal gynecologic wounds.

Single reports present effectiveness of NPWT combined with split-thickness skin grafting after surgical treatment of rare diseases of the vulva such as Paget’s disease, hidradenitis suppurativa or syringoid eccrine carcinoma [61–63].

NPWT was shown to be effective in preventing surgical site infections (SSIs) in women after cesarean section. Administration of single-use NPWT for 7 days postoperatively in 50 patients prevented SSIs and consequently readmissions to the hospital in the high-risk group of women with BMI ≥35 kg/m^2^ [64]. In a retrospective cohort study conducted by Mark et al. including 63 patients after cesarean section with BMI of >45 kg/m^2^, the use of NPWT reduced the percentage of wound complications from 10.4 % in the control group to 0 % in the study group (*p* = 0.15) [65]. On the other hand, significantly longer duration of surgery and lower percentage of scheduled cesarean sections were observed in the control group, possibly contributing to the higher rate of complications.

Single attempts were made to use subatmospheric pressure in the treatment of necrotising fasciitis in women after cesarean section [66, 67]. As a result of a complex management strategy including surgical debridement of necrotic tissue, use of broad-spectrum antibiotics with simultaneous negative pressure therapy wounds were completely healed in two patients who had been diagnosed with this potentially life-threatening infection in the postoperative period [66, 67].

A team of Danish researchers presented interesting study regarding psychosocial aspects of NPWT among patients in an outpatient setting [68]. Based on an analysis and interpretation of individually collected interviews of 10 patients with wound healing disorders including four patients after cesarean section i.a. the efficacy of therapy and its impact on everyday functioning, need for relatives care and support, and ability to manage a device were assessed. The study indicated that in general, patients considered NPWT to be effective, and despite the fact that it was associated with a feeling of dependence at the beginning, therapy became more acceptable with time. Importantly, all patients reported a feeling of embarrassment during social situations due to a device being present. With regard to patients after cesarean section who at the same time comprised a group of young mothers, help and support of their relatives were extremely important.

Finally, single reports analyzing the costs of vacuum therapy in obstetrics and gynecology suggest economic benefits of the treatment. A study conducted by Lewis et al. using the theoretical model to assess the costs of care using prophylactic NPWT in a group of 431 patients after laparotomy due to gynecological malignancy revealed the cost-effectiveness of such management amounting to $104 savings per one patient compared to the routine management with the assumption of 50 % treatment efficacy [69]. The generated savings were even higher in the group of obese and morbidly obese patients; in authors’ opinion, this group of patients may benefit most from prophylactic NPWT.

Despite unquestionable benefits of NPWT it is necessary to pay special attention to iatrogenic mistakes that might occur during treatment as evidenced by Beral et al. who reported a case of abnormal wound healing as a consequence of retained foam pieces in patient after total abdominal hysterectomy [70]. Taking into account radiolucent nature of the foam making subsequent detection difficult as well as the fact that often many fragments are used to fill in the wound bed it seems reasonable to record the number of removed foam pieces during each dressing replacement.

Summarizing all written above, the review of available literature leads to a conclusion that negative pressure wound therapy constitutes a promising alternative to the standard wound management regimens in obstetrics and gynecology. Vacuum therapy appears to be particularly beneficial in the group of obese patients, patients undergoing radical vulvectomy, vulvar reconstruction and patients with history of radiation therapy. In women after cesarean section and with risk factors responsible for abnormal wound healing, prophylactic NPWT may prevent surgical site infections and additional hospital stay, reduce treatment costs and, what is equally important, permit the patient to fulfill her role as a mother without any restrictions. Similar as in the case of growth factors, there are not enough randomized controlled trials to justify the use of NPWT in everyday clinical practice while simultaneously analyzing the effect of the vacuum therapy on the overall treatment costs. It is necessary to develop a unified regimen for the use of NPWT, defining the optimum negative pressure levels, dressing type, dressing change intervals and treatment duration depending on the type of the wound. It also appears reasonable to explore the possibilities of using NPWT in combination with other wound treatment methods, such as growth factors or antiseptic dressings.

## Silver dressings—state-of-the-art antiseptic dressings in obstetrical and gynecological practice

Infections are one of the main factors responsible for impaired postoperative wound healing. In the conditions of intact integuments integrity, the epidermis acts as a mechanical barrier against pathogenic microorganisms supported by the acidic environment and physiological bacterial flora on the skin surface. However, as the skin is incised, these mechanisms of protection lose their relevance and the wound becomes an open gate for pathogens. Endo- and exotoxins produced by microorganisms alter the course of healing, simultaneously depleting local environment of oxygen and nutrients. The inflow of inflammatory cells into the wound, stimulated by the presence of pathogens crucial in the initial phase of healing, may further enhance hypoxia, inhibit the activity of growth factors and extend the overall healing time if the infection prolongs. As a result, a negative feedback loop is activated, with oxygen deficiency causing tissue necrosis within the wound and promoting growth of pathogenic microorganisms. In addition, depletion of oxygen impairs the host cell-mediated response of leukocytes and makes the local microenvironment prone to colonization by anaerobic bacteria [71].

The risk factors for the surgical site infection are similar to the factors impairing normal wound healing process as discussed in introduction and include: elderly age, obesity, diabetes, malnutrition, anemia, nicotinism, renal and liver impairment, immunosuppression, irradiation as well as the size, depth and location of the wound, duration of the surgery, type of suturing materials used, presence of drains, damage and hypoperfusion of the surrounding tissues, free spaces left and insufficient hemostasis [5, 71, 72]. Hair shaving, particularly on the day before the procedure, is responsible for the increased percentage of SSIs, as is prolonged hospitalization [2, 5, 72]. Since the risk factors of wound infection are similar to factors responsible for disturbances in normal healing process, it appears reasonable to treat every case of a chronic, difficult-healing wound as potentially infected.

According to the guidelines of the Centers for Disease Control and Prevention, postoperative wounds in obstetrics and gynecology are classified as clean-contaminated [72]. Literature data estimate the incidence of infected wounds in obstetrics and gynecology at 1–4 % to 8–12 % [1, 7, 10–12]. With regard to the two most common procedures—abdominal hysterectomy and cesarean section, SSIs rates are 3.0–12.2 % and 1.8–11.3 %, respectively, while in women after surgical treatment of cancer of the vulva, the percentage of wound infections is even greater and amounts to 21–39 % [1–5, 7, 8, 10–13].

In most cases, microorganisms responsible for the infections of obstetric and gynecological postoperative wounds are the patient’s endogenous bacterial flora. Most commonly isolated strains include: *Staphylococcus aureus*, aerobic Gram-negative bacilli (*Escherichia coli*, *Proteus* sp., *Klebsiella* sp., *Enterobacter* sp.), *Enterococcus* sp., β-hemolyzing streptococci of groups A, B, C and G, anaerobic bacterial species and *Pseudomonas aeruginosa* [1, 7, 10, 11]. Methicillin-resistant *Staphylococcus aureus* (MRSA) is detected in 2–53 % inoculates from infected obstetric/gynecological wounds [7, 10, 11]. Fungi, mainly *Candida* sp. constitute a rare etiological factor in postoperative wound infections in gynecology [7].

Proper management of infected wounds is a multistage process involving wound debridement, lavasepsis and the use of local and/or systemic agents (antiseptics, antibiotics). In the era of increasing bacterial resistance to antibiotics, topical treatment with antiseptics plays an important role, as the agents are less selective but allow to achieve higher therapeutic concentrations within the wound, particularly in concomitant ischemic conditions. Antiseptic dressings are an example of such activity; among these, dressings containing silver are the group of best documented efficacy.

Antiseptic properties of silver in the treatment of wound infections were already known in the ancient times. Today, silver dressings are a novel method for topical treatment of infected and difficult-to-heal wounds. This is mostly due to the silver’s broad spectrum of antimicrobial action against both fungi and bacteria including MRSA or vancomycin-resistant *enterococci* (VRE) [20, 71, 73–77]. Combined with relatively low toxicity, aforementioned properties make silver a very valuable tool for fighting pathogens responsible for infections of wounds after iatrogenic activities.

The mechanisms of silver action involve inhibition of the cellular respiration, binding of nucleic acids and causing their denaturation, inhibiting cell replication and altering the permeability of microbial cell membranes [20, 71, 73, 74, 78]. This is achieved by means of reactions of the silver ions with proteins, DNA or RNA and negatively charged chloride ions inside pathogens cells. An adverse side of this interaction is the inactivation of highly reactive and positively charged silver ions (Ag^+^) by chlorides and various anionic complexes present in the wound bed. As a result, a rapid drop in the concentration of an active form of silver that might effectively inhibit the growth of microorganisms responsible for the infection occurs within the wound. According to the literature data, concentrations of silver associated with the highest bactericidal efficacy as measured by the 3-log reduction in the bacterial counts should exceed 30–40 mg/l [20, 71, 73]. Therefore, silver-based treatment of infected wounds requires that the dressings provide appropriate concentrations of Ag ions in the wound bed and maintain these concentrations for possibly the longest time, thus ensuring adequate activity and preventing resistance.

For nearly four decades of their use, silver nitrate and silver sulfadiazine became gold standards in the silver-based treatment of wound infections [71]. Both products contain positively charged Ag ions in high concentrations (0.5 % silver nitrate solution—3176 mg/l; 1 % silver sulfadiazine—3025 mg/l) [20, 71]. Although, concentration values markedly exceed the recommended levels of 30–40 mg/l, due to the presence of Ag^+^, both drugs are characterized by low residual activity [20, 71, 73]. Achieving appropriate antimicrobial activity requires, therefore, frequent drug applications into the wound region—for silver sulfadiazine, it is recommended to change the dressing twice a day while for silver nitrate, dressings should be changed 12 times during each 24 h [20, 71, 73, 74].

An innovation in the silver-based therapy of infected wounds—nanocrystalline silver dressings were introduced into clinical use in the late 1990s. The novelty of these dressings as compared to the dressings discussed above consists in releasing both positively charged Ag ions and uncharged Ag (Ag^0^) forms [20, 71, 73–75]. Since uncharged silver is less prone to react with anionic complexes, it is possible to maintain appropriate concentration and activity of silver inside the wound for longer periods. As the reserves of ionic silver are depleted, additional amounts of Ag^0^ and Ag^+^ ions are released from the dressing, ensuring continuous and steady supply of active silver [73]. The clinical implication of these properties is the ability to change the dressing less frequently, resulting in the treatment being more comfortable to the patient and protecting the wound from injuries that might occur upon the dressing change [20, 71, 73, 74]. Contrary to other types of dressings where silver is added in the form of a solution, cream, ointment or an additional dressing layer, incorporation of silver nanocrystals with the diameters of <20 nm into the dressing facilitates accumulation of larger quantities of silver within a small volume. In practice this allows to achieve high initial concentration of silver within the wound. In case of nanocrystalline silver dressings, this concentration is 70–100 mg/l and may be maintained at this level for up to 7 days [20, 71, 73, 75].

The superiority of nanocrystalline silver over silver nitrate and silver sulfadiazine in inhibiting bacterial growth was demonstrated by Yin et al. [76]. Following inoculation of dressings with an aliquot of bacterial suspension to reach approximately 10^7^ colony-forming units of *S. aureus*, researchers demonstrated that the use of nanocrystalline silver was able to reduce the bacterial counts to less than 10^2^ cells after 1 h application. In case of silver nitrate and silver sulfadiazine, similar results were obtained after 4 and 6 h, respectively. Study conducted in 1998 by Wright et al. evaluated bactericidal effects of silver nitrate, silver sulfadiazine and nanocrystalline silver against particularly resistant strains, such as MRSA and VRE [77]. Using a methodology similar as the previously mentioned investigators, the authors observed a 7-log reduction in MRSA and VRE counts following 30 min after inoculation when nanocrystalline silver was used. Reduction of such magnitude could not be observed for dressings containing silver nitrate or silver sulfadiazine after incubation lasting three and half hours. Interestingly, both drugs showed only minor antibacterial activity after 30 min of incubation.

Apart from antimicrobial activity silver delivery systems, in particular silver nanoparticles present anti-inflammatory properties depending on the delivery technique, available concentration of silver and duration of release. Reduction in matrix metalloproteinases’ (MMPs) levels is one of the actions of particular importance as it was demonstrated that the release of metalloproteinases 2 (MMP-2) and 9 (MMP-9) while being indispensable for normal healing, may alter its course when present at excess concentrations by degrading fibronectin, vitronectin and peptide growth factors [71, 73–75, 78]. The remaining properties of nanocrystalline silver are responsible for the down-regulation of inflammatory activity within the wound area by reducing the TNF-α production and inducing apoptosis [71, 73–75, 78].

Among the reports published to date on the use of silver-containing dressings in the treatment of infected postoperative wounds, only a few were based on randomized controlled trials conducted in appropriately large subject groups with majority being in vitro or case series studies. Number of reports describing the use of silver in the treatment of infected and hard-to-heal postsurgical wounds in obstetrics and gynecology is also limited.

Markowska-Sioma conducted a prospective study to assess the efficacy of metallic-coated silver dressings in the treatment of difficult-healing wounds after major gynecological surgeries [79]. During a 10-month follow-up period, healing disorders were observed in two patients after radical vulvectomy and in one patient after abdominal hysterectomy. Bacteriological examination of wounds revealed the presence of *Pseudomonas aeruginosa*, *Proteus mirabilis*, *Streptococcus anhaemoliticus* and *Enterococcus faecalis* in patients after radical vulvectomy, while a negative result of culture was obtained for patient after abdominal hysterectomy. An initial stage of the wound treatment included autolytic debridement followed by cleansing of the wound with octenidine before each dressing change. At the beginning of the treatment silver dressings were changed every day, and after clinical improvement every 2–3 days. On day 14, complete healing of abdominal hysterectomy wound was observed; radical vulvectomy wounds took longer to heal, with no times of complete healing being indicated in the article. In patients’ opinion, the treatment outcomes were positive.

Prophylactic use of silver dressings for the prevention of surgical site infections in women undergoing cesarean section was the subject of one full text study. Connery et al. conducted a retrospective study assessing the efficacy of dressings consisting of nylon fibers autocatalytically coated with metallic silver for the prevention of SSIs after the cesarean section [80]. Among 72 patients in the study, 36 were included in the control group and managed in conventional manner using gauze pads. In the follow-up period, postsurgical wound infections were observed in two patients in the study group and two patients in the control group. The obtained results could not demonstrate that silver-impregnated dressings significantly reduced the risk of SSIs following cesarean section; however, due to the fact that comorbidities were significantly more common in the study group, the obtained results might, in the opinion of investigators, not fully reflect the efficacy of the tested dressings.

In summary, silver dressings may comprise a useful tool in the treatment of infected obstetric and gynecological wounds, although only limited reports suggest their beneficial effect on the healing process in both wounds following vulvectomy and wounds after laparotomy as part of hysterectomy or cesarean section procedures. The proven efficacy of silver is largely due to its low toxicity and broad spectrum of antimicrobial action, which is particularly important in the era of increasing bacterial resistance to antibiotics. On the other hand, recently published studies on the prevention of wound infections in patients undergoing cesarean section did not confirm a higher efficacy of silver dressings compared with standard dressings while pointing out the high cost of such treatment. As a consequence, similar as in the case of NPWT and growth factors, a higher number of prospective studies must be conducted in an appropriately large population of women to develop standardized management methods making use of individual silver dressings, especially with regard to the particularly beneficial nanocrystalline silver dressings.

## Summary

Obstetrics and gynecology are examples of surgical disciplines where wound healing disorders are one of the most common complications and the efficacy of standard treatment regimens is not always satisfactory. Chronic, difficult-to-heal and infected wounds lead to the development of further, oftentimes serious complications that reduce the quality of life of the patients, extend hospitalization times and generate additional treatment costs. Conclusions from the literature review regarding the use of growth factors, NPWT and silver dressings suggest that these methods may play an important role in the management of wound after invasive obstetric and gynecological procedures. This is particularly relevant in a group of high-risk patients, e.g., obese patients, patients after vulvectomy or previous radiation therapy. The use of novel wound management methods in above-mentioned patient cohorts may significantly improve the final treatment outcomes, reduce postoperative complications and the related morbidity and mortality rates, improve the quality of life and reduce costs due to additional medical procedures. On the other hand, the non-numerous studies evaluating first attempts to use these methods in obstetrics and gynecology, mostly non-randomized and conducted in small populations, do not permit any definite conclusion regarding their usefulness, efficacy and cost-effectiveness. Prospective studies on the use of growth factors, platelet-rich plasma derivatives, negative pressure wound therapy and silver dressings in the treatment of chronic, hard-to-heal postsurgical wounds following obstetric and gynecological interventions are required.
